# Clinical and immunological characteristics of five patients with immune dysregulation, polyendocrinopathy, enteropathy, X-linked syndrome in China–expanding the atypical phenotypes

**DOI:** 10.3389/fimmu.2022.972746

**Published:** 2022-08-24

**Authors:** Yu Huang, Shuyu Fang, Ting Zeng, Junjie Chen, Lu Yang, Gan Sun, Rongxin Dai, Yunfei An, Xuemei Tang, Ying Dou, Xiaodong Zhao, Lina Zhou

**Affiliations:** ^1^ National Clinical Research Center for Child Health and Disorders, Ministry of Education Key Laboratory of Child Development and Disorders, Chongqing Key Laboratory of Child Infection and Immunity, Children’s Hospital of Chongqing Medical University, Chongqing, China; ^2^ Department of Hematological Oncology, Children’s Hospital of Chongqing Medical University, Chongqing, China; ^3^ Department of Rheumatism and Immunology, Children’s Hospital of Chongqing Medical University, Chongqing, China

**Keywords:** IPEX syndrome, primary immunodeficiency disease, *FOXP3* mutations, regulatory T cells, atypical phenotypes

## Abstract

**Background:**

Immune dysregulation, polyendocrinopathy, enteropathy, X-linked (IPEX) syndrome is a rare disorder of the immune regulatory system caused by forkhead box P3 (*FOXP3*) mutations. Abnormal numbers or functions of regulatory T (Treg) cells account for the various autoimmune symptoms. We aimed to explore the molecular genetics and phenotypic spectra of patients with atypical IPEX syndrome in China.

**Methods:**

We analyzed the molecular, clinical and immune phenotype characteristics of five Chinese patients with *FOXP3* mutations.

**Results:**

We summarized the molecular and phenotypic features of five patients with *FOXP3* mutations, including two novel mutations. Four of the five patients displayed atypical phenotypes, and one developed immune-related peripheral neuropathy. Three of the five patients showed normal frequencies of Treg cells, but the proportions of subsets of Treg cells, CD4^+^ T cells and B cells were out of balance.

**Conclusions:**

Our report broadens the understanding of the clinical features of atypical IPEX syndrome. Our detailed analyses of the immunological characteristics of these patients enhance the understanding of the possible mechanisms underlying the clinical manifestations.

## Introduction

Immune dysregulation, polyendocrinopathy, enteropathy, X-linked (IPEX) syndrome (OMIM #304790) is a rare monogenic primary immunodeficiency disease caused by mutations of the X-linked gene forkhead box P3 (*FOXP3*) (1–3). IPEX syndrome is characterized by the classical triad of early-onset intractable enteropathy, type 1 diabetes mellitus (T1DM) and chronic dermatitis. Other autoimmune symptoms can also develop, such as hypothyroidism and antibody-mediated cytopenia (4–6). IPEX patients who do not receive treatments mostly fail to thrive and die within the early years of life (7). Allogeneic hematopoietic stem cell transplantation (HSCT) and immunosuppression (IS) are currently available, proven treatments (8).

The incidence of IPEX syndrome is less than one in a million, and to date, more than seventy pathogenic mutations of *FOXP3* have been described in over 200 patients (6, 7, 9–12). The correlation of *FOXP3* genotype and clinical phenotype in IPEX syndrome is not straightforward because the same mutation in different individuals may result in different clinical presentations (7, 13). With the development of gene sequencing technology, an increasing number of atypical cases of IPEX syndrome have been reported, including cases with late-onset symptoms, single-organ involvement, mild disease phenotypes or rare clinical features (14, 15). Patients with such atypical symptoms are often first misdiagnosed before a correct diagnosis is made.

Here, we report the clinical and immunologic features of five patients from China with different *FOXP3* mutations, including two novel mutations. Among the five patients, four presented with atypical phenotypes; one suffered from immune-related peripheral neuropathies, which have rarely been reported (16); and three had normal percentages of regulatory T (Treg) cells but significantly elevated CD25^-^Foxp3^+^CD4^+^ T-cell (CD25^-^Foxp3^+^) levels relative to the healthy controls.

## Materials and methods

### Patients

The patients enrolled in this study were suspected to have mutations in *FOXP3*. All patients were admitted to the Children’s Hospital of Chongqing Medical University. Hemizygous *FOXP3* mutations were identified in five patients using high-throughput sequencing. Informed consent was obtained from the patients’ guardians. This study was conducted in accordance with the tenets of the Declaration of Helsinki and was approved by the ethics committee of Children’s Hospital of Chongqing Medical University.

### PCR for Sanger sequencing

Genomic DNA was extracted from whole blood cells using the QIAamp DNA Mini Kit (Qiagen, Germany) according to the manufacturer’s instructions, and then polymerase chain reaction (PCR) was performed to amplify *Foxp3*. The primer sequences used to amplify the mutations in patient 2 (P2), P3, and P4 were F: 5′-TCCCCATTCAGAGCATTGAGC-3′ and R: 5′-AATGTGAGGTTAGGTTCCCTGC-3′. The primer sequences used to amplify the mutation in P5 were F: 5′-CCCAGAACAACCATACCCACA-3′ and R: 5′-ATCCCGTTCCTCCCCAATGT-3′. F and R represent forward and reverse, respectively. The PCR products were sequenced by Sangon Biotech (Shanghai, China).

### Immunophenotyping

Immunophenotyping of lymphocyte subpopulations was performed with the following antibodies: CD3-PerCP (clone: HIT3a, BioLegend), CD4-FITC (clone: RPA-T4, BioLegend), CD8-BV510 (clone: RPA-T8, BioLegend), CD45RA-PE-Cy7 (clone: HI100, BioLegend), CD27-APC (clone: M-T271, BioLegend), TCR aβ-PE (clone: IP2b, BioLegend), TCR γδ-BV421 (clone: B1, BioLegend), CD19-APC (clone: HIB19, BioLegend), CD27-V450 (clone: M-T271, BioLegend), IgD-AF488 (clone: IA6-2, BioLegend), CD24-PE (clone: ML5, BioLegend), and CD38-PerC P (clone: HIT2, BioLegend). The samples were acquired on a FACSCanto II flow cytometer, and the data were analyzed by FlowJo. All reference values were obtained from our recent study on peripheral lymphocyte phenotyping (17).

### Flow cytometric analysis of CD4^+^ T-cell and B-cell subsets

To analyze Treg cells, peripheral blood mononuclear cells (PBMCs) were first stained with CD4-PE-Cy7 (clone: RPA-T4, BioLegend), CD25-BV421 (clone: BC96, BioLegend), and CD45RA-FITC (clone: HI100, BD Biosciences) antibodies. Next, the cells were fixed and permeabilized using the eBioscience Intracellular Fixation and Permeabilization kit (Thermo Fisher Scientific). Finally, the cells were stained with FOXP3-PE (clone: PCH101, eBioscience/ThermoFisher Scientific) and CD152-APC (clone: BNI3, BD Biosciences) antibodies.

Circulating follicular helper T (cTfh) cells, circulating follicular regulatory T (cTfr) cells, subsets of T helper (Th) cells and cTfh cells were quantified in 50 μL whole blood in three separate experiments. Whole blood cells were stained with CD3-PerCP (clone: HIT3a, BioLegend), CD4-PE-Cy7 (clone: RPA-T4, BioLegend), CD45RO-APC (clone: UCHL1, BD Biosciences), CD45RA-FITC (clone: HI100, BD Biosciences), CXCR5-BV421 (clone: J25ID4, BioLegend), CD25-APC (clone: MT271, BioLegend), CD127-PE (clone: A019D5, BioLegend), CXCR3-APC (clone: 1C6, BD Biosciences), and CCR6-PE (clone: G034E3, BioLegend) antibodies for 30 min at 4°C.

For characterization of circulating B-cell subsets, PBMCs were stained with CD19-PerCP-Cy5.5 (clone: SJ25C1, BioLegend), CD27-PE-Cy7 (clone: MT271, BioLegend), and IgM-APC (clone: G20-127, BD Biosciences) antibodies. The samples were acquired on a FACSCanto II flow cytometer, and the data were analyzed by FlowJo.

### Conservation and pathogenicity analysis of mutated amino acids

Conservation analysis of amino acid sequences was performed by Clustal Omega (https://www.ebi.ac.uk/Tools/msa/clustalo/). Pathogenicity analysis of mutated amino acids was performed by MutationTaster (http://www.mutationtaster.org/), PolyPhen-2 (http://genetics.bwh.harvard.edu/pph2), Provean (http://provean.jcvi.org/index.php), SIFT (http://sift.jcvi.org), and Fathmm (http://fathmm.biocompute.org.uk).

### Structural analysis of Foxp3

The crystal structure of Foxp3 (PDB: 4wk8), which was determined by X-ray diffraction at a resolution of 3.40 Å, was used as the template. The structural impact of mutations was analyzed by PyMOL. The mutant residues and certain nearby residues are depicted. For a clear demonstration of the interresidue relationship, some residues are highlighted in specific colors with the computed labeled hydrogen bonds.

### Statistical analysis

Data were analyzed using the unpaired *t* test. All statistical analyses were conducted in GraphPad Prism 8 software (GraphPad Software, Inc., San Diego, CA). P ≤ 0.05 was considered to indicate a significant difference.

## Results

### Clinical characteristics

**Figure 1:**
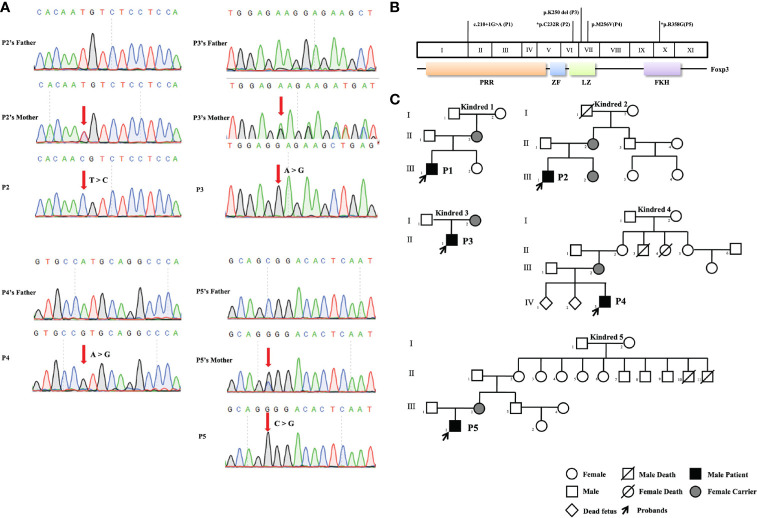
Pedigrees and pathogenic *FOXP3* mutations of the five patients. **(A)** Sanger sequencing confirmed the hemizygous *FOXP3* mutations of the patients. The red arrows indicate the locations of mutations. **(B)** The *FOXP3* coding region is shown, with the pathogenic mutations carried by the five patients in this study. *Refers to novel mutations. The other mutations have been previously reported. **(C)** Pedigrees of the five families with *FOXP3* mutations. Each family is designated by a number (1 to 5). Black indicates patients, and the probands are indicated by arrows.

Five *FOXP3* mutations were identified in five patients by next-generation gene sequencing and confirmed by Sanger sequencing (**Figure 1A**). One (the mutation in P1) of the five mutations was located in the proline rich N-terminal (PRR) domain of FOXP3, three (the mutations in P2, P3, and P4) were located in the leucine zipper (LZ) domain, and one (the mutation in P5) was found in the C-terminal FKH domain (**Figure 1B**). The patients were from five different families, and all of them were male. In all kindreds, the mothers of the patients were carriers. In particular, P4’s mother had two miscarriages (IV 1, 2) for unknown reasons. In kindred 5, II10 died of diarrhea (**Figure 1C**).

**Figure 2:**
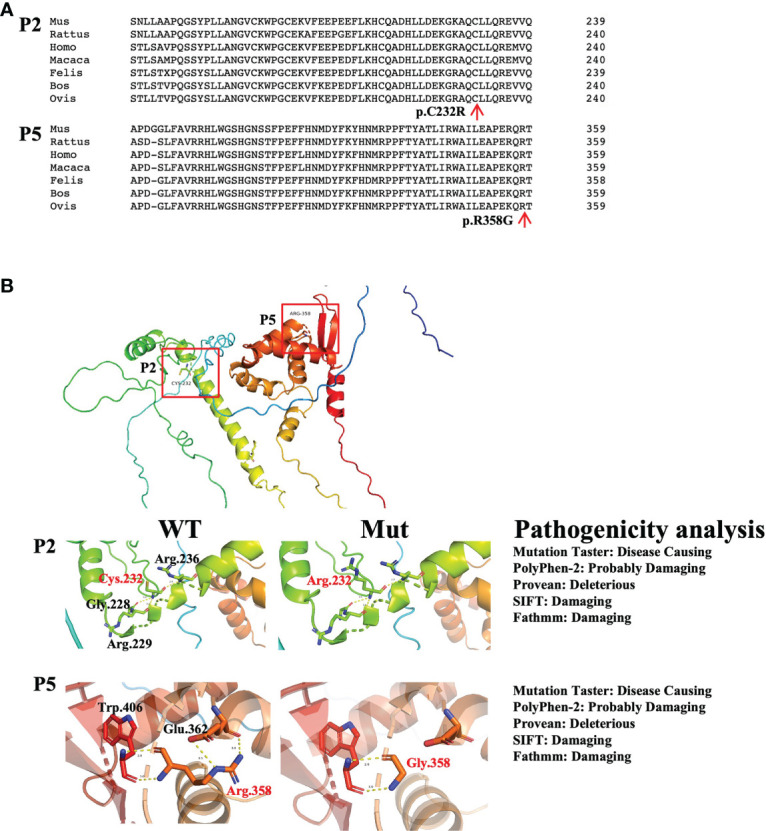
Pathogenicity analysis of the novel mutations. **(A)** Conserved analysis of amino acid sequences in different species by Clustal Omega. The red arrows refer to mutated amino acid sites. **(B)** The left panel shows the structural impacts of the mutations on the FoxP3 structure (PDB: 4wk8), which were analyzed by PyMOL. Hydrogen bonds are shown as dotted lines. Red indicates mutated amino acids, and black indicates amino acids around the mutations. The right panel shows the results of the pathogenicity analysis of novel mutations by MutationTaster, PolyPhen-2, Provean, SIFT and Fathmm.

There were two novel mutations: the mutation in P2 (c.694T>C, p.C232R) and the mutation in P5 (c.1072C>G, p.R358G). For the novel mutations, we performed pathogenicity analysis. The two mutated amino acids were highly conserved on multiple sequence alignment (**Figure 2A**) and predicted to cause disease by several algorithms (MutationTaster, PolyPhen-2, Provean, SIFT, and Fathmm) (**Figure 2B**). Structural analysis of the p.R358G mutant showed that the replacement of hydrophilic arginine with hydrophobic glycine disrupted the formation of hydrogen bonds with glutamate. As a result, the mutation may destabilize the protein structure or directly disrupt the functional site. The p.C232R mutation did not affect the hydrogen bonds between the amino acids (**Figure 2B**).

The clinical characteristics of the patients are summarized in **Table 1**. The five patients were aged from 5 y 2 m to 14 y 5 m, and of note, the age of onset of three patients (P1, P2, and P4) was after 4 y. Diabetes was the initial symptom in P2 and P4, while the remaining patients first presented with diarrhea (P1), eczema (P5) and anemia (P3). As the disease progressed, P3 developed severe and refractory diarrhea, P1 developed chronic gastritis with eosinophil infiltration, and P5 presented with chronic diarrhea. Two patients had thyroid-related complications, including chronic thyroiditis (P2) and hypothyroidism (P4). In addition, P5 was diagnosed with congenital thyroid deficiency when he was born, which may occur *in utero*. Dermatitis was found in all the patients except for P2. Autoimmune hemolytic anemia was found in P1 and P3; additionally, P3 had thrombocytopenia. Three patients (P1, P2, and P3) presented with lymphadenopathy, and P1 also had hepatosplenomegaly. Failure to thrive and pneumonia were common in all the patients, and P3 had a concurrent severe fungal infection.

**Table 1:** Summary of the clinical, immunologic, and molecular findings in patients with IPEX syndrome.

	P1	P2*	P3	P4	P5*
**Sex**	Male	Male	Male	Male	Male
**Mutation**	c.210+1G>A	c.694T>C	c.748_c.750delAAG	c.766A>G	c.1072C>G
		p.C232R	p.K250del	p.M256V	p.R358G
**Age of onset**	5y1m	7y	2m	4y2m	2m
**Symptoms at onset**	Diarrhea	Diabetes	Anemia	Diabetes	Eczema
**Treg**	Decrease	Normal	Decrease	Normal	Normal
**Enteropathy**	Chronic diarrhea Chronic gastritis with eosinophil infiltration	_	Severe chronic diarrhea	_	Chronic diarrhea
**Endocrinopathy**	_	Diabetes Chronic thyroiditis	Diabetes	Diabetes Hypothyroidism	Congenital thyroid deficiency
**Skin disease**	Dermatitis	_	Dermatitis	Dermatitis	Dermatitis
**AID**	Autoimmune hemolytic anemia	_	Autoimmune hemolytic anemia Thrombocytopenia	_	_
**Other**	Lymphoadenopathy Hepatosplenomegaly Allergic asthma Pneumonia Failure to thrive	Lymphoadenopathy Failure to thrive	Lymphoadenopathy Nephropathy Fungal infection Thrush Pneumonia Food allergy Failure to thrive	Membranous nephropathy Pneumonia Coombs neg.anemia Failure to thrive Immune-associated peripheral neuropathy	Pneumonia Otitis media Food allergy Allergic conjunctivitis Failure to thrive
**Autoantibodies**	ANA(1:100)	a-TG(1358) a-TPO(>2000)	_	Ro-52(++) a-TG(48.4) a-TPO(11) GAD(48.4) NF 155(1:10) a-GD3(+) a-GT1a(+)	_
**Immune globulin**	IgG 20.4g/L IgA 4.42g/L↑ IgM 122g/L IgE 6510IU/ml↑	IgG 12.4g/L IgA 3.49g/L IgM 0.492g/L IgE 52.3IU/ml	IgG 9.42g/L IgA 1.65g/L IgM 3.85g/L IgE 23.1IU/ml	IgG 5.75g/L IgA 1.31g/L IgM 0.449g/L↓ IgE 326IU/ml↑	IgG 12.2g/L IgA 0.186g/L↓ IgM 0.744g/L IgE 1042IU/ml↑
**Eosinophilia(cells/uL)**	650↑	410	170	370	430
**Treatment**	Rapamycin/Prednisone	MMF	Rapamycin/Prednisone Cyclosporin A/HSCT	Prednisone/MMF/Rapamycin	Prednisone/MMF
**Outcome**	Alive/10y9m	Alive/15y8m	Death/5y2m	Alive/7y5m	Alive/7y

ANA, antinuclear antibody; MMF, mycophenolate mofetil; HSCT, hematopoietic stem cell transplantation; a-TG, thyroglobulin antibody, a-TPO, thyroid peroxidase antibody; GAD glutamic acid decarboxylase antibody; NF 155, neurofascin 155; a-GD3, gangliosides D3 antibody; a-GT1a, gangliosides T1a antibody; -, didn't test.

Surprisingly, P4 experienced gross motor skill regression at the age of 6 y 9 m. Neurofascin 155, anti-ganglioside D3 antibody and anti-ganglioside T1a antibody were detected in his serum. No obvious abnormalities were found through either electroencephalogram or cranial MRI. These examinations indicated dysfunction of motor and sensory conduction. Thus, P4 was diagnosed with immune-associated peripheral neuropathy at the age of 7 y.

Immunosuppressors such as rapamycin and mycophenolate mofetil were used in all of the patients, and four of the five patients (except P2) received prednisone treatment. P3 received cyclosporin A and hematopoietic stem cell transplantation but died of severe bowel rejection, while the remaining patients’ symptoms were controllable to a certain degree.

### Laboratory findings

Some laboratory findings are presented in **Table 1**. Immunoglobulin analyses revealed that P5 had decreased IgA levels, and he developed relatively severe respiratory infections. Three of the five patients (P1, P4, and P5) showed elevated IgE levels, and P1 also had eosinophilia. Different autoimmune antibodies were found in P1, P2 and P4. P2 and P4 had autoantibodies affecting the thyroid, and related clinical symptoms were also present. Some autoimmune peripheral neuropathy-related antibodies were detected in P4, as described above.

### Evaluation of Treg-cell subsets

**Figure 3:**
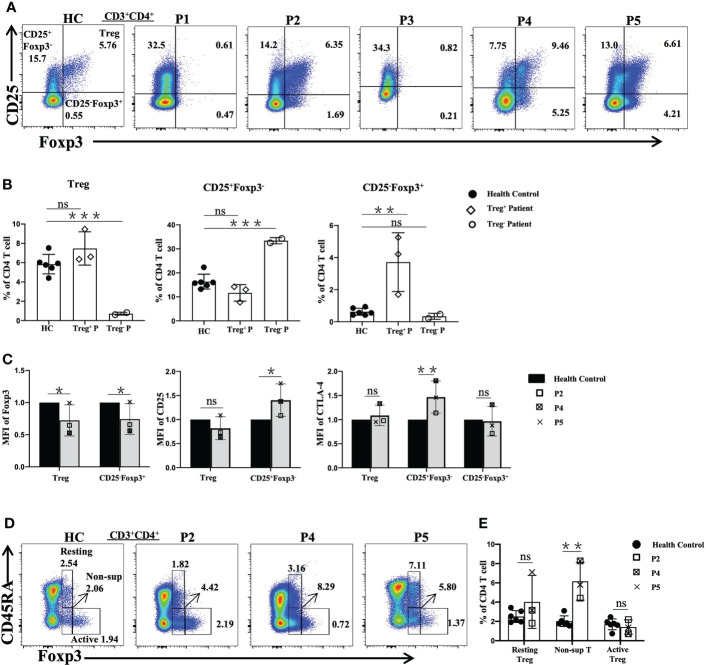
Flow cytometric analysis of Treg-cell subsets in patients with FOXP3 mutations. **(A)** and **(B)** The percentages of Treg (CD4^+^CD25^+^Foxp3^+^), CD25^+^Foxp3^-^CD4^+^ T and CD25^+^Foxp3^-^CD4^+^ T cells among CD4^+^ T cells. **(C)** The mean fluorescence index of Foxp3, CD25 and CTLA-4 in different cells. **(D)** and **(E)** The percentages of Treg-cell subsets, namely, resting Treg (CD45RA^+^Foxp3^+^), non-suppressive T (CD45RA^-^Foxp3^+^) and active Treg (CD45RA^-^Foxp3^++^) cells among CD4^+^ T cells. Treg^+^ Patient, patient with normal numbers of Treg cells; Treg^-^ Patient, patient without Treg cells. *P<0.05; **P<0.01; ***P<0.001; ns, no significance.

Flow cytometric analysis revealed that three patients (P2, P4, and P5) showed normal frequencies of Treg cells (**Figure 3A**), but higher (P<0.01) percentages of CD25^-^Foxp3^+^ cells were observed relative to the age-matched normal controls (**Figure 3B**).

Furthermore, the mean fluorescence intensity (MFI) values of FOXP3, CD25 and cytotoxic T lymphocyte-associated protein 4 (CTLA-4) in Treg, CD25^+^Foxp3^-^CD4^+^T (CD25^+^Foxp3^-^) and CD25^-^Foxp3^+^ cells were assessed by flow cytometry in the patients with normal Treg cells and the normal controls (**Figure 3C**). Compared with the values in the normal controls, the FOXP3 MFI values of Treg cells and CD25^-^Foxp3^+^ cells were lower, while the CD25 MFI value was higher (P<0.05) in the CD25^+^Foxp3^-^ cells of the patients, but there was no difference in Treg cells. The MFI value of surface CTLA-4 of Treg and CD25^-^Foxp3^+^ cells of the patients was similar to that of the normal controls, while the MFI value of CTLA-4 of CD25^+^Foxp3^-^ cells was higher in the patients than in the healthy controls (P<0.01).

Treg cells were subdivided into three subpopulations based on the expression of Foxp3 and CD45RA, including resting Treg cells (CD45RA^+^Foxp3^low^), active Treg cells (CD45RA^-^Foxp3^high^) and non-suppressive T cells (CD45RA^-^Foxp3^low^) (18). Compared to the normal controls, P2, P4 and P5 showed significantly increased percentages of non-suppressive T cells, while the percentages of active Treg cells of P4 and P5 were reduced. However, P2 showed relatively normal resting Treg and active Treg cell proportions (**Figures 3D, E**).

### Skewing of peripheral T-cell subsets

Peripheral blood lymphocyte subsets were analyzed in all the patients (**Table 2**). When the tests were performed, all the patients were in the active phase of autoimmune disease and P3 also had pneumonia. Only two (P2 and P5) of the five patients received immunosuppressive therapy, and P1 was treated with prednisone. P2 and P4 showed slightly increased proportions of T cells, while the frequency of T cells in P3 was decreased. P5 displayed an increased percentage of CD8^+^ T cells and CD4^+^ T cells. Further analyses of T-cell subsets revealed that P2 had an increased proportion of naive CD8^+^ T cells. However, the percentages and numbers of central memory CD8^+^ T cells, effector memory CD8^+^ T cells, effector memory CD4^+^ T cells, TCRαβ^+^ double-negative T cells and natural killer (NK) cells in P2 were decreased. The proportions of central memory CD4^+^ T cells and TCRαβ^+^ double-negative T cells were higher in P3 than in the healthy controls. However, the ratios of CD4 naive T cells and γδ T cells in P3 were decreased. The percentage and number of NK cells in P5 were slightly higher than those in the normal controls.

**Table 2:** Lymphocyte classification of patients with IPEX syndrome.

	P1		P2		P3		P4		P5	
	Relative (%)	Absolute(cells/ul)	Relative (%)	Absolute(cells/ul)	Relative (%)	Absolute (cells/ul)	Relative (%)	Absolute(cells/ul)	Relative (%)	Absolute (cells/ul)
T cell	77.30 (55-78)	3339.4 (1325-2276)	79.6 (56.84-75.02)	1473.0 (1184-2144)	52.3 (60.05-74.08)	2945.6 (1424-2664)	84.25 (55-78)	1769.8 (700-4200)	68.5 (53.88-72.87)	4004.9 (1794-4247)
CD8^+^T cell	30.04 (19-34)	1298.0 (480-1112)	33.9 (21.91-36.80)	627.3(489-1009)	21.8 (19.68-34.06)	1229.0 (518-1125)	32.01 (19-34)	672.4 (300-1800)	33.8 (19.00-32.52)	1793.6 (580-1735)
CD4^+^T cell	38.43 (27-53)	1660.5 (531-1110)	38.8 (22.25-39.00)	717.8 (522-1084)	27.4 (26.17-40.76)	1542.1 (686-1358)	46.41 (27-53)	974.8 (300-2000)	52.5 (24.08-42.52)	1842.8 (902-2253)
B cell	18.95 (10-31)	818.8 (216-536)	12.8 (8.84-17.76)	236.6 (203-476)	31.1 (10.21-20.12)	1752.1 (280-623)	8.26 (10-31)	173.5 (200-1600)	9.0 (13.23-26.39)	528.3 (461-1456)
NK cell	3.59 (4-26)	155.1 (246-792)	7.4 (10.12-28.34)	136.3 (210-804)	15.4 (9.00-22.24)	864.2 (258-727)	7.44 (4-26)	156.38 (90-900)	21.17 (7.21-20.90)	1238.4 (270-1053)
CD8 Naive	_	_	84.4 (35.34-72.32)	529.5 (231-568)	53.0 (41.58-77.90)	651.4 (297-730)	_	_	65.0 (36.80-83.16)	1165.8 (356-1095)
CD8 TEMRA	_	_	6.8 (5.08-31.24)	42.7 (29-269)	13.1 (1.70-24.62)	161.0 (11-218)	_	_	14.5 (0.84-33.02)	260.1 (9-440)
CD8 CM	_	_	7.8 (10.96-31.00)	48.6 (74-228)	28.2 (12.08-30.54)	346.6 (85-268)	_	_	18.3 (5.18-31.66)	328.2 (56-406)
CD8 EM	_	_	1.0 (2.38-15.84)	6.5 (16-109)	5.7 (1.58-13.18)	69.8 (10-129)	_	_	2.2 (0.70-11.22)	39.8 (6-145)
CD4 Naive	_	_	61.3 (39.50-66.26)	440.0 (230-627)	43.9 (45.56-75.28)	677.0 (321-972)	_	_	54.5 (46.14-84.40)	1004.3 (472-1760)
CD4 TEMRA	_	_	0.7 (0.00-1.54)	5.1 (0-12)	0.2 (0.00-1.06)	3.5 (0-13)	_	_	1.1 (0.00-1.36)	19.7 (0-22)
CD4 CM	_	_	34.5 (25.34-49.90)	247.6 (182-403)	53.7 (22.06-46.46)	828.1 (211-478)	_	_	41.9 (13.88-48.12)	772.1 (212-735)
CD4 EM	_	_	3.6 (4.68-15.70)	25.8 (29-117)	2.2 (2.08-8.78)	34.1 (23-84)	_	_	2.5 (0.94-6.46)	46.4 (15-87)
TCRαβ^+^DNT	_	_	0.5 (0.61-2.31)	8.0 (12-37)	3.0 (0.18-2.81)	88.4 (4-55)	_	_	1.5 (0.37-1.80)	61.6 (9-57)
γδ T cell	_	_	8.1 (6.55-20.28)	118.6 (81-343)	3.1 (6.92-19.84)	90.1 (124-410)	_	_	13.5 (4.94-17.98)	540.7 (114-539)
Memory B	_	_	63.1 (7.15-23.10)	149.3 (20-86)	8.0 (7.76-19.90)	140.0 (31-94)	_	_	5.0 (2.98-14.18)	26.6 (26-124)
Naive B	_	_	7.2 (53.78-78.64)	16.9 (116-347)	83.2 (48.36-75.84)	1457.7 (147-431)	_	_	77.2 (65.54-86.62)	407.8 (323-1089)
Transitional B	_	_	1.1 (1.38-9.42)	2.5 (4-37)	0.6 (2.58-12.30)	10.9 (10-66)	_	_	0.79 (5.24-17.22)	4.2 (35-172)
Plasmablasts B	_	_	5.0 (0.49-7.06)	11.7 (1-23.0)	2.0 (0.90-7.36)	34.9 (4-28)	_	_	1.5 (0.50-7.06)	8.0 (4-63)
CD4:CD8	1.28 (0.98-1.94)		1.14 (0.65-1.65)		1.25 (0.87-1.94)		1.45 (0.98-1.94)		1.0 (0.90-2.13)	

-, didn't test.

**Figure 4:**
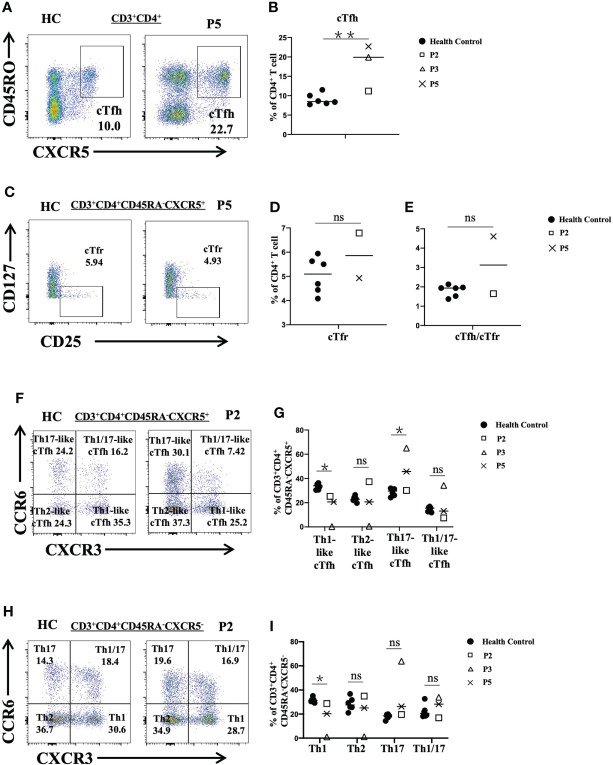
Flow cytometric analysis of peripheral CD4^+^ T-cell subsets in patients with FOXP3 deficiency. **(A)** and **(B)** The percentage of cTfh cells (CD3^+^CD4^+^CD45RA^-^CXCR5^+^) among CD4^+^ T cells. **(C)** and **(D)** The frequency of cTfr cells (CD3^+^CD4^+^CD127^-^CD25^+^) among CD4^+^ T cells. **(E)** The ratio of cTfh cells to cTfr cells. **(F)** and **(G)** The percentages of cTfh-cell subsets: Th1-like cTfh cells (CXCR3^+^CCR6^-^), Th2-like cTfh cells (CXCR3^-^CCR6^-^), and Th17-like cTfh cells (CXCR3^-^CCR6^+^). **(H)** and **(I)** The frequency of CD4^+^ T-cell (CD3^+^CD4^+^CD45RA^-^CXCR5^-^) subsets: Th1 cells (CXCR3^+^CCR6^-^), Th2 cells (CXCR3^-^CCR6^-^), and Th17 cells (CXCR3^-^CCR6^+^). *P<0.05; **P<0.01; ns, no significance.

In particular, we analyzed different subsets of CD4^+^ T cells in P2, P3 and P5. We observed that the proportions of cTfh cells in P3 and P5 were increased (P<0.01) (**Figures 4A, B**) and that the frequency of cTfr cells in P2 was higher than that in the healthy controls (**Figures 4C, D**). The ratio of cTfh cells to cTfr cells in P5 was higher than that in healthy controls (**Figure 4E**). Further analysis of cTfh-cell subsets showed that the levels of Th17-like cTfh and Th1/17-like cTfh cells in P3 were increased. However, the levels of Th1-like cTfh cells in P2, P3 and P5 were reduced (P<0.05). The level of Th2-like cTfh cells was also reduced in P3 but was increased in P2 (**Figures 4F, G**). When we analyzed CD4^+^ T subsets according to cytokine secretion, we found that P3 had higher proportions of Th17 cells and Th1/17 cells, but the ratios of Th1 and Th2 cells were decreased compared to those in the normal controls. P5 showed increased Th17-cell and reduced Th1-cell levels (**Figures 4H, I**).

### Abnormal B-cell phenotypes

**Figure 5:**
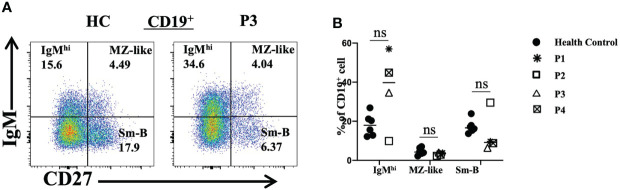
Flow cytometric analysis of peripheral B-cell subsets in patients with FOXP3 deficiency. **(A)** and **(B)** The percentages of IgM^hi^ B cells (CD19^+^IgM^+^CD27^-^), MZ-like B cells (CD19^+^IgM^+^CD27^+^) and smB cells (CD19^+^IgM^-^CD27^+^) among B cells *P<0.05; ns, no significance.

We also analyzed peripheral blood B lymphocyte subsets. The total frequencies and numbers of B cells were increased in P3, while the frequencies of B cells in P4 and P5 were decreased. Further analyses revealed that the frequencies and numbers of memory B cells in P2 and the frequencies and numbers of naive B cells in P3 were higher than the reference values. However, the levels of naive B cells in P2 and the levels of transitional B cells in P2, P3 and P5 were reduced (**Table 2**). Furthermore, P1, P3 and P4 had increased proportions of IgM^hi^ B cells and decreased proportions of switched memory B (smB) cells (**Figures 5A, B**). P2 had decreased frequencies of IgM^hi^ B cells and marginal zone-like B (MZ-like) cells, but the proportion of smB cells was increased (**Figure 5B**).

## Discussion

In this study, we described in detail the clinical and immunological features of five patients with different *FOXP3* mutations. The *FOXP3* gene encodes the Foxp3 protein, which is essential for the development and regulatory function of Treg cells (19, 20). Treg cells are often involved in maintaining tolerance, preventing inflammatory diseases, regulating immunity against infections, restraining tumor and anti-autoimmune responses, and so on (21).

As the clinical manifestations of IPEX patients vary, it is not surprising that four of our 5 patients (except P3) showed atypical clinical features, including unreported symptoms, late-onset of the disease, a single system affected and, unusually, the absence of severe early-onset diarrhea in our patients. Decreased numbers of Treg cells are often reported in IPEX patients, but in our study, three patients showed normal numbers of Treg cells and aberrant levels of Treg-cell subsets.

Early-onset intractable enteropathy, T1DM and chronic dermatitis are the classical triad of IPEX syndrome. However, four of our patients (except P3) only developed one of these symptoms as the main symptom and none of them had early-onset intractable diarrhea. In particular, late onset of symptoms was found in three patients (P1, P2, and P4), as most of the IPEX patients developed symptoms within 1 year of age. Three of the five mutations identified in these patients have been reported before (6, 22), but the clinical manifestations and examination results were not identical.

In particular, P4 developed immune-related peripheral neuropathy in the course of the disease, which has rarely been reported (16). P4 had the same mutation as one newly reported patient with atypical late-onset severe gastritis. The previously reported patient had autoimmune hepatitis and gastritis as his main clinical symptoms, while P4 showed severe diabetes and nephropathy (22). As the disease progressed, P4 showed gross motor skill regression and was diagnosed with immune-associated peripheral neuropathy, as related examinations indicated dysfunction of motor and sensory conduction. Neurological disease in IPEX patients has rarely been reported, except in one patient with progressive cerebellar atrophy, which is not considered to be associated with immune dysfunction (16).

The c.210+1G>A (P1) mutation has been described in detail in three patients; all of them had kidney involvement, but our patient did not. The autoimmune diseases of the reported patients included T1DM, thyroiditis and hepatitis (23, 24), but the most prominent symptom in our patient (P1) was autoimmune hemolytic anemia. Therefore, atypical clinical manifestations and different clinical manifestations caused by the same mutation may delay the diagnosis and treatment of the disease.

Two of the five mutations identified in our study had not been previously reported, and the two patients with these mutations showed atypical manifestations. The mutation in P2 was located in the LZ domain of FOXP3; disruption of the LZ domain is predicted to block transcriptional regulation of target genes and may be associated with severe autoimmune manifestations (25). As a result, P2 developed T1DM and chronic thyroiditis as the main clinical symptoms. The FKH domain interacts with many other proteins and assists in Treg transcription, but it does not affect the numbers of Treg cells (25). Hence, mutations in the FKH domain are probably associated with a milder clinical phenotype, as we found that dermatitis was the main feature of P5. Interestingly, P5 showed congenital thyroid deficiency, which has not been reported before.

The frequencies of Treg cells in three of our patients were normal, but these patients showed relatively decreased frequencies of CD25^+^Foxp3^-^ cells and obviously increased frequencies of CD25^-^Foxp3^+^ cells. CD25^-^Foxp3^+^ cells lose their immunosuppressive function (26), which might explain the autoimmune manifestations of these three patients. In addition, the expression of Foxp3 in Treg and CD25^-^Foxp3^+^ cells were decreased, which can explain the loss of suppressive function and contribute to the clinical features. Treg cells usually highly expressed CD25. We found that the expression of CD25 was decreased in two of the three patients with Treg cells. As previous studies have shown, CD25 can absorb IL-2 as a cytokine sink, which deprives conventional CD4^+^ T cells of IL-2 and thereby contributes to the suppressive function of Treg cells. As a result, conventional CD4^+^ T cells are restricted from expanding and differentiating into effector T cells (6, 27). Thus, decreased expression of CD25 in mutated Treg cells may disrupt their function and differentiation.

Resting Treg cells are a stable Treg-cell phenotype, and active Treg cells have high suppressive activity. Non-suppressive T cells lack suppressive activity and can secrete inflammatory cytokines such as IL-2 and IFN-γ; thus, the majority of these cells act as activated T cells (18). In our study, P4 and P5 showed imbalances in Treg subsets. Thus, we speculate that the functions of Treg cells were disrupted in P4 and P5, although the cell numbers were in the normal range, and the increased number of non-suppressive T cells may also have contributed to the autoimmune disease the patients experienced. In addition, an increase in non-suppressive T cell numbers can lead to the production of more IL-2, which may in turn reduce the expression of CD25.

Treg cells can gain an effector phenotype concomitant with loss of Foxp3 expression. These cells may adapt to their environment and gain expression of transcription factors and chemokine receptors. Phenotypic changes in cells are normally associated with Th1, Th2, Th17, and cTfh cells (28). Previous studies have indicated that cTfh cells are associated with humoral autoimmunity and are found in several monogenic immunodeficiencies (29–31). P3 and P5 had elevated levels of cTfh cells, and they both had severe autoimmune diseases. When CD4^+^ T- and cTfh-cell subsets were analyzed, only P3 displayed extreme subset skewing. P3 showed elevated Th17-cell and Th17-like-cTfh cell levels. Th17 cells can secrete IL-17, which is highly associated with enteritis (32), and this may account for P3’s intractable diarrhea. cTfr cells constitute approximately 30% of non-suppressive T cells in healthy people (33). Thus, the increased proportion of cTfr cells in P2 along with elevated non-suppressive T cells might account for the loss of function of Treg cells.

A previous study revealed that some patients showed increased levels of transitional B cells and naive B cells (34); however, detailed analysis of B-cell subsets in three patients showed decreased levels of transitional B cells and inconsistent changes in naive B cells and memory B cells. cTfh cells offer critical help in B-cell class switching and affinity maturation (35), and two of the three patients showed an increased cTfh-cell frequency. Thus, we speculate that the autoimmune diseases of these patients may have influenced the cTfh-cell balance and B-cell tolerance. A more in-depth assessment of B-cell compartments may resolve these conflicting findings.

IS and HSCT are the main treatments for IPEX patients. Four of our patients received IS-inducing therapy, including MMF and rapamycin. Only P3 received HSCT for severe enteropathy, but unfortunately, he died of severe graft-versus-host-disease. P1 and P5 did not develop autoimmune disease after administration of IS-inducing treatment and prednisone. However, previous studies indicate that receipt of IS-inducing therapy or HSCT does not influence overall survival; however, less organ impairment before HSCT is related to a higher chance of survival (8). In addition, although current immunosuppressive regimens have limitations, rapamycin has been demonstrated to be the most beneficial IS-inducing therapy (36–38).

In conclusion, we described the molecular, clinical, and immune characteristics of five patients with *FOXP3* mutations. We report the molecular genetics, clinical heterogeneity, and immunological abnormalities of IPEX patients, which may help us improve the diagnosis and treatment of patients with atypical manifestations. However, due to the limited number of cases, the mechanisms underlying the heterogeneity of clinical features, immune abnormalities and outcomes need to be further studied.

## Data availability statement

The original contributions presented in the study are included in the article/supplementary files. Further inquiries can be directed to the corresponding authors.

## Ethics statement

The studies involving human participants were reviewed and approved by Institutional Review Board of Children’s Hospital of Chongqing Medical University. Written informed consent to participate in this study was provided by the participants’ legal guardian/next of kin.

## Author contributions

YH, LZ, and XZ designed experiments and analyzed the data. YH wrote the first draft of the manuscript and performed the experiments. SF, TZ, JC, LY, and GS assisted with basic immunological analysis. RD, YA, XT, and YD contributed to scientific discussion, data interpretation, and revision of the manuscript. LZ and XZ supervised the research and revised the manuscript. All authors contributed to the article and approved the submitted version.

## Funding

This work was supported by the General Basic Research Project from the Ministry of Education Key Laboratory of Child Development and Disorders (No. GBRP-202119).

## Acknowledgments

We thank the patients and their families for participating in this study. We also thank the doctors, nurses, and other health care providers at the Children’s Hospital of Chongqing Medical University.

## Conflict of interest

The authors declare that the research was conducted in the absence of any commercial or financial relationships that could be construed as a potential conflict of interest.

## Publisher’s note

All claims expressed in this article are solely those of the authors and do not necessarily represent those of their affiliated organizations, or those of the publisher, the editors and the reviewers. Any product that may be evaluated in this article, or claim that may be made by its manufacturer, is not guaranteed or endorsed by the publisher.
